# Adolescents’ understanding of obesity: a qualitative study from rural South Africa

**DOI:** 10.1080/16549716.2021.1968598

**Published:** 2021-09-06

**Authors:** Tshegofatso M. Seabi, Ryan G. Wagner, Shane A. Norris, Stephen M. Tollman, Rhian Twine, David B. Dunger, Kathleen Kahn

**Affiliations:** a Medical Research Council/ University of the Witwatersrand Rural Public Health and Health Transitions Research Unit (Agincourt), School of Public Health, Faculty of Health Sciences, University of the Witwatersrand, Johannesburg, South Africa; b Samrc Developmental Pathways for Health Research Unit, Department of Paediatrics, School of Clinical Medicine, University of the Witwatersrand, Johannesburg, South Africa; c Department of Paedaitrics, Wellcome Trust-MRC Institute of Metabolic Sciences, University of Cambridge, Cambridge, UK

**Keywords:** Body mass index, perceptions, overweight, young people, Sub-Saharan Africa

## Abstract

**Background:**

Levels of obesity are rising in South Africa, notably among adolescent females. Excessive energy-dense diets and physical inactivity are among the factors contributing to this increase. Given that these factors are largely behavioural, understanding young people’s views of obesity can contribute to more targeted behavioural interventions. Yet little is known of how rural South African adolescents view obesity.

**Objectives:**

The aim of this study was to explore rural South African adolescents’ views of obesity, including their understanding of its causes, consequences, and solutions.

**Methods:**

This qualitative study took place within the MRC/Wits Rural Public Health and Health Transitions Research Unit (Agincourt) study area, in rural northeast South Africa. Three focus group discussions were held with male (n = 16) and female adolescents (n = 15), aged 14–19 years in 2018. Data were analysed using thematic analysis and the Social Cognitive Theory used to frame the findings.

**Results:**

Participants presented conflicting views of obesity, with both positive and negative opinions expressed. Causes of obesity were seen to be multifactorial, including genetics, diet, lack of physical activity, and HIV treatment. Adolescents proposed medication and hospitalisation as ways to address obesity. When discussing interventions to address obesity, adolescents expressed the need for more information, suggesting that providing information to both themselves and their family members as part of interventions would be important.

**Conclusions:**

Rural South African adolescents have a complex perspective of obesity, likely driven in part by the current nutrition transition underway and do not inherently see individual behaviour as a driver or mitigator of obesity. Complex interventions including the involvement of other household members are needed to change adolescents’ views on the role of the individual, and ultimately, change both individual and household behaviour to prevent obesity.

## Background

Sub-Saharan Africa is predicted to experience an increasing number of adolescents in the coming years [1], despite having the worst adolescent health profile globally [2,3]. High levels of mortality from maternal and infectious causes (including HIV [3]) persist and are coupled with increasing behavioural risks for non-communicable diseases (NCDs), including high levels of tobacco and alcohol use, low levels of physical activity [4], poor diet and increasing levels of overweight and obesity [5,6]. In studies from rural South Africa (SA) and other parts of sub-Saharan Africa (SSA) [7–9], the double burden of malnutrition, particularly stunting, in the early years of life, coupled with increasing overweight and obesity in adolescent girls and young women, is evident [9]. To mitigate the growing epidemic of obesity, understanding adolescents’ knowledge and views of obesity is needed to tailor interventions aimed at changing behaviour to reduce levels of obesity.

Obesity is a complex, multifactorial and usually preventable condition that results from a combination of individual, social, community and societal-level determinants [10]. Traditionally defined as a body-mass index (BMI) >30, the prevalence of obesity continues to rise globally, a trend reflected in SSA [11], where levels of obesity differ significantly between males and females, with South African females seeing a rapid rise during adolescence [8,9]. Childhood and adolescent obesity have serious consequences for health and social wellbeing in later life and it is widely agreed that obesity in these age groups should be reduced [12,13]. As part of the reducing childhood obesity mandate, understanding obesity from the perspective of this age group is important for developing context-specific interventions.

The behaviour change approach promotes health through individual lifestyle changes that are appropriate to one’s unique context [14]. The approach posits that before individuals are able to change their lifestyles, they must first understand basic facts about a particular health issue, adopt key attitudes, learn a set of skills and be given access to appropriate services [15]. Fundamentally, it is believed that people do not resist change but resist being changed [15]. Thus, the approach promotes health collaboratively, tailored to an individual’s specific context. To provide individuals with information to enact behaviour change, it is important to understand both the knowledge base that individuals currently have and the context from which their views emerge. This has formed the basis for this study: to develop an understanding of rural SA adolescents’ views of obesity. The Social Cognitive Theory (SCT) is one theory that has been used to understand behaviour and behaviour change in order to plan and asses interventions [16]. It has been used in planning of various interventions such as Healthy Relationships, an intervention that is specifically designed to engage with persons living with HIV/AIDS directly addressing prevention of transmission to others [17,18]. In this current paper, SCT will be used to frame the findings.

Few studies from either rural or urban South Africa have explored views and understanding of obesity. Studies from urban Soweto have specifically examined body image and satisfaction, body shapes, eating habits and attitudes mostly among female adolescents [19–21]. They concluded that South African female adolescents are struggling with body image and desire ‘unhealthy’ mostly related to bigger body shapes, with the majority of participants having a distorted body image [22–25]. While these studies examined body image, they fell short of exploring reasons for the adolescents’ understanding of the causes of obesity and did not discuss possible interventions to change BMI levels. Furthermore, adolescent males and rural participants have been largely excluded from these types of studies.

Due to the paucity of empirical data, we set out to explore rural South African adolescents’ views of obesity, including their understanding of its causes, consequences, and solutions. Views are defined for the purpose of this study as reported attitudes, opinions, beliefs, feelings, understandings and experiences of adolescents in relation to obesity [26].

## Methods

### Study setting

This work was undertaken at the MRC/Wits Rural Public Health and Health Transitions Research Unit (Agincourt) study area, located in rural Mpumalanga Province, northeast South Africa close to the Mozambique border. The Unit runs the Agincourt Health and Socio-Demographic Surveillance System (HDSS) which has accumulated over 25 years of comprehensive surveillance data on a population of some 116, 000 people living in 31 villages in 2018. The resident adolescent population aged 14–19 in 2018 numbered 14,154, of which 50.7% were female.

### Study sample

This study design is a qualitative phenomenology approach. The focus of the approach is on experience from the perspective of the individual, their personal knowledge and subjectivity, and emphasises the importance of personal perspective and interpretation. As such it is useful for understanding subjective experience and gaining insights into individual’s motivations and actions. Qualitative data were collected during focus group discussions (FGDs). Participants for the FGDs were conveniently recruited from one village within the Agincourt HDSS study area. Using tracing information from the Agincourt HDSS, households with adolescents were visited by trained fieldworkers who invited adolescents and their caregivers to participate. Adolescents, for the purpose of this study, were defined as males and females 14–19 years of age at the time of enrolment. For adolescents younger than 18 years, caregiver consent and adolescent assent was sought. For participants older than 18 years, adolescent consent alone was sought. Consent and assent were conducted in the local language (xiTsonga).

## Data collection

The groups of 8–10 individuals were constituted by age and sex: one all-male, one all-female and one mixed male/female group (Table 1). The differing compositions aimed to respond to the dynamic that a group with same gender participants are more likely to express themselves freely, while in mixed sex group it was expected that different perspectives would arise. FGDs were chosen to allow for a less intimidating environment than one-on-one interviews and to allow for discussion between participants. Participants chose the day and group that they wanted to participate in, with the FGDs carried out at a central venue within the village. Trained FGD facilitators who invited them to participate and consent, also reminded them of the focus groups on the day before the scheduled sessions. FGD facilitators (one male and one female) were trained on the guide [27]. Both FGD facilitators had a secondary school qualification, previous experience working in the community as fieldworkers and were fluent in both English and xiTsonga. The FGDs averaged 50 minutes (ranging between 45 and 50 minutes in the three groups). After undertaking three (3) FGDs, saturation was reached on the key elements of the points of discussion. Saturation was determined by undertaking debriefing sessions and analysing the transcripts for emergence of new themes after each group session. Table 2 shows the guiding questions that were posed to the focus group participants.

**Table 1.** Number and ages of participants in the FGD groups

	Mixed Sex FGD	All male FGD	All female FGD
Number of Participants	9 (5 girls, 4 boys)	12	10
Age range (in years)	14–18	16–19	15–19

**Table 2.** Guiding questions for the focus group discussions

**Personal/Cognitive Factors** Sometimes, people refer to others as ‘obese’. What do you understand by this? What are your thoughts and feelings about food? What are your thoughts and feelings about exercise? We are interested in factors that lead to people being light or heavy. What do you think are reasons that some people are heavy, and others are light? In your views, what do you think the best size and shape is for young people? And why? **Behaviour** What food do you eat? What food is eaten by the adolescents in your community? Would they be considered healthy or unhealthy? What exercises do you do? **Environmental Factors**
What are some of the things that you have heard or seen about obesity in young people?
What are some of the consequences of obesity that you have heard about? Do you think your chances of being overweight or obese is very high when parents and/or family are overweight and/or obese? Are there other factors? **Closing/overall question**: Thank you for your participation, lastly, what do you think can be done or what is your wish to happen to reduce obesity amongst adolescents?

## Data analysis

The FGDs explored rural adolescents’ understanding of overweight and obesity, particularly causes and consequences on health, using a semi-structured guide (Table 2). FGDs were conducted in xiTsonga and audio recorded. They were then transcribed and translated into English by the FGD facilitators. Thematic analysis allows for flexibility in analysis and the summary of key themes of data. Overall, inductive thematic analysis was conducted to capture themes that emerged from the collected data [27]. The inductive approach allowed initially for data driven analysis without fitting into a framework. We then presented the data using the social cognitive theory as a framework. This framework was selected after reviewing the themes that emerged from the inductive thematic analysis and is used to provide structure to the results section.

To ensure trustworthiness, in the preparation phase, the study was presented to a team of colleagues to interrogate the methods, sampling, interview guide and analysis plans, with several iterations of input and translations. After the data were transcribed and translated, transcripts were cleaned and checked to ensure that the transcripts were representative of the focus group discussions. To address bias and ensure rigour, the transcripts were coded independently by a second researcher and themes were identified and validated to agreement.

## Theoretical framework

The Social Cognitive theory (SCT) provides a framework for understanding, predicting and changing human behaviour [28]. Developed from social learning theory, SCT offers a comprehensive framework for understanding health-related behaviours [29]. The key SCT construct of reciprocal determinism means that an individual is understood to be both an agent for change and a responder to change. Thus, changes in the environment, and reinforcements, can be used to promote healthier behaviour [16].

**Figure 1.**
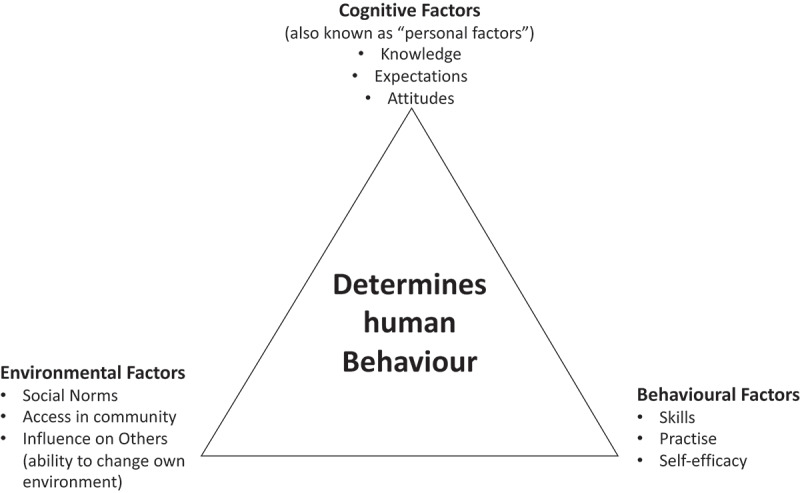
Illustration of the three main factors of the social cognitive theory [16]

The three primary factors of SCT are as follows: firstly, behavioural factors which include an individual’s ability to perform the behaviour when desired and obtain desired outcomes, as well as the confidence that one can perform a specific behaviour under a variety of circumstances. Secondly, environmental factors such as learning how to do a behaviour by watching someone do it and receiving reinforcement for it, such as community/ family norms and access [29]. Lastly, are cognitive factors; these focus on an individual’s knowledge, expectations and thought processes. The primary driver for behaviour change interventions using the SCT model is acknowledgement of the bidirectionality of these three key elements (Figure 1) [30], highlighting the individual’s control of his/her own behaviour. Individuals can achieve self-control by setting specific behavioural change goals, monitoring their own behaviour through the process of change, and making changes as needed [16]. This paper uses the SCT to discuss and interpret the findings of rural South African adolescents’ views on obesity. The SCT is used as a lens through which to start to understand the development of obesity interventions for rural adolescents.

## Ethics

The study was approved by the Human Research Ethics Committee (Medical) of the University of Witwatersrand (M171113, M180873), and the Mpumalanga Department of Health Research Committee. Permission from village leadership was obtained through established processes of the Public Engagement Office of the MRC/Wits Rural Public Health and Health Transitions Research Unit (Agincourt).

## Results

Thirty-one adolescents consented to participate in the FGDs. The average age was 16 years (range: 16–18 years). All participants resided in the same village, and the average BMI for the FGD participants was 20.8 for males and 22.5 for females (both considered to be normal BMI values [31]).

## Main findings from the FGDs

As noted above, we undertook an inductive thematic analysis approach and present the findings under the three main constructs of the SCT to provide structure. The three constructs are as follows: cognitive factors, environmental factors, and behavioural factors. What follows are the three SCT constructs supported by the inductive themes that emerged from the analysis and accompanying quotes from the focus group discussions. The results as presented address the adolescents’ views including their understanding of the causes and consequence of obesity, and their proposed solutions.

### Cognitive factors

Overall, adolescents expressed conflicting views on obesity, with both males and females providing positive and negative views fitting the cognitive/personal factors of the SCT model. The juxtaposing positive and negative views expressed highlight the complex knowledge and attitudes towards obesity that adolescents in the context have.

Positive views included the suggestion that obese people were happy and without problems.

“ … Like happiness can make you fat … ” (P1_all female FGD)

“ … Some people become fat if they don’t have problems … ” (P2_mixed FGD)

Whilst others negatively suggested that obese people were lazy.

“ … In most cases people who are fat or obese or overweight are lazy … ” (P1_all male FGD)

### Environmental factors

The SCT defines environmental factors as encompassing learned behaviour and reinforcement from the environment relating to community/family norms and access. In this theme, adolescents identified immediate environment (family) and medication and disease and suggest ways to address obesity through environmental means.

### Immediate environmental factors

Family environment was mentioned as another likely contributor to obesity. Participants expressed that obese adolescents often resided with other obese family members. This related to the acceptance of appearance or social norms within the adolescent’s immediate environment. This sentiment was shared by most participants. However, some participants, particularly male participants, disagreed, noting that individuals can have different body compositions when compared to their relatives.

Adolescents also suggested that, in addition to the immediate family environment, genetics could be involved.

Because there was a time where I was seeing myself as very thin and I tried to eat a lot as I was thinking that I will be fatter than they [referring to family members] are but it didn’t work. It doesn’t matter as to whether I eat lot of fatty foods or what, I am always like this (P6_all male FGD)

I think that it also depends on how you want your body to be like because when I look at both my parents, they look like they can’t manage their body because they are fat. So, it is better for me to stay the way I look now so that I can be able to do my own things (P7_all male FGD)

#### Medication and disease

In addition to diet, physical activity and environmental factors, adolescents mentioned the use of some medications, particularly contraceptives and HIV medication, as causes of obesity. The all-female group highlighted the link between contraception (or oral *prevention* as it was referred to) and obesity, suggesting that some females that were obese were so not because of diet, but because they were on oral or injectable contraceptives. Participants expressed the belief that HIV treatment caused obesity as well. Some adolescents described it as ‘being liked by the treatment’ while another related it to the medication causing an increased intake of food.

And nowadays we are preventing, in the mobile [clinic], the injection can make you fat. The medication they are taking … and Prevention [contraception] (P3_ all female FGD) … HIV … if you took treatment correctly and don’t skip or miss you can be fat (P4_ all female FGD)

Adolescents also mentioned the consequences of obesity, particularly physical impairment and diseases like diabetes, hypertension, and heart disease.

Some people when they are obese, when they grow up you find that they are no longer able to walk (P1_all female FGD) … And they have some diseases, heart attack (P3_all female FGD) … sugar diabetes and high blood [pressure]

#### Tackling obesity through environmental means

When asked about how to reduce obesity, adolescents described obesity as an illness or disease that needs to be treated. They mentioned multiple ways to address obesity in their community: amongst of which are focusing on restricting access to outlets selling unhealthy foods and providing access to facilities to tackle obesity such as closure of fast-food outlets. Adolescents also mention unhealthy ways that adolescents have addressed obesity including the use of substances.

Participants suggested closure of fast-food outlets that sell unhealthy food as an intervention. Participants externalised responsibility for obesity to food outlets in their community rather than their own choices and practices. They particularly mentioned shops owned by other ethnic groups as problematic. The suggestion is that adolescents may not view their behaviour or actions as a solution to overweight/obesity, but that targeting environmental factors, such as fast-food outlets, were more effective interventions for reducing obesity, thus not claiming responsibility for their own choice of buying and consuming food from those shops.

“ … We need to shut down the Indian shops in the community … They are selling us things that are not healthy, and they don’t even eat those things, but they want us to eat them … ” (P6_all male FGD)

Adolescents offer a justification for the use of substances such as marijuana (dagga) to reduce obesity. While acknowledging its use increases the likelihood of risky behaviour, adolescents mentioned these substances are helpful in dealing with psychosocial challenges such as stress, humiliation from peers, and bullying, as well as weight loss. Participants described it more as self-medication to address obesity than as a substance abuse issue amongst overweight and obese adolescents. This highlights the interplay between personal factors, behaviour, and the availability of these substances.

“ … So, emotionally people can laugh at you to an extent that you will feel sad about it … people finally smoke dagga or marijuana because they want to get slim (P6_all male FGD) … ”

“ … Other people go to smoke dagga/marijuana thinking that it will make them lose weight because they are stressed that other people will laugh at them … ” (P2_all female FGD)

### Behavioural factors

Rural South African adolescents’ views on obesity reflect a complex and multi-causal picture that ultimately results in adverse effects on individuals. Some ways to address obesity have been discussed above; however, in addition adolescents viewed some behavioural factors such as diet and location as well as physical activity as contributors to obesity. They also address behavioural factors that should be included in the solutions, including the provision of skills and knowledge, creating opportunities for self-efficacy, and creating opportunities to influence their environment through training and knowledge.

#### Diet and location

Adolescents identified diet as a significant cause of obesity, discussing what they believed to be healthy and unhealthy food choices and how food decisions relate to obesity. Adolescents identified vegetables, such as leafy green ‘morogo’ grown in the community and fruit as healthy food and identified ‘kotas’ (made up of white bread, green mangoes fermented in oil and spices, processed meat, deep-fried chips and sometimes cheese) and ‘vetkoek’ (deep-fried dough), sweets or candy and crisps, as unhealthy food. Adolescents mentioned that excessive eating of unhealthy, oily foods caused obesity in their community.

In addition to describing unhealthy diet as a contributor to obesity, adolescents further differentiated the locations of where healthy and unhealthy foods were consumed. They mentioned that at school (referring to the school feeding program) and at home they ate what they considered to be healthy food, while on school trips and with their peers within the community, they ate what they considered to be unhealthy food, suggesting that the ability access healthy or unhealthy is related to location and peer presence.

[At school] beans, samp [African food consisting of dried corn kernels that have been stamped and chopped until broken], rice, milk and at home we eat Nkanka, bangala [traditional vegetables] (P7_all male FGD) xiendla hi vomu [corn and crushed nuts] tihove [samp, ground nuts and crushed nuts] and porridge (P8_all male FGD)

Just like when you have gone out with friends or maybe you went on a trip, you would want to buy food that is a bit expensive depending on how much they have given you at home. That’s when we go to Debonairs or KFC [fast food outlets] but when we are here at home [talking about when they are with their peers in the community], we eat chips and cold drinks [referring to eating with friends] (P6_all female FGD)

#### Physical activity

Physical activity, in the form of exercise or lack thereof, was also mentioned as a cause of obesity. There were different forms of physical activity that adolescents described, from walking to school daily and house chores, to male adolescents playing soccer. Undertaking physical activity seemed to be more strongly related to the ability to defend or protect oneself in the community for male adolescents and less about being healthy. The adolescent males prefaced the discussion with ‘*these days it is not about exercising’* referring to their belief that it’s not about only being healthy. Adolescent males also expressed common use of steroids to lose weight, build muscle and defend themselves.

Steroids are pills or the juice that we drink that makes you very fit or have muscles … It causes your body to develop faster than the way it was supposed to … When they are busy provoking him, you find that they just hit him with a fist and run away and another one comes and does the same and runs away, so he must be fit to run after them (P6_ all male FGD) …

In the all-female group, while lack of physical inactivity was mentioned as a cause of obesity, it seemed to be considered less important, and causing discomfort.

others don’t consider it [referring to exercise] as important (P8_all female FGD) … I don’t do it because when you do it once, when you wake up in the morning you need to do it again and if not, your knees can be painful (P6_all female FGD)

#### Hospitalisation and medications

Participants suggested that obese individuals should be hospitalised or given medication, pills, or injections to help them reduce weight. The discourse around helping obese or overweight individuals regarding hospitalising them reiterates the prevailing sense that adolescents do not immediately identify themselves as agents capable of impacting their own BMI. Most participants viewed obesity as a disease or a reflection of disease, and therefore a condition treated clinically.

“ … I was thinking that it will be better if they can build a hospital for the obese people so that they can … fix them, they give them some injectables or tablets, things like that … or make them exercise … ” (P7_all male FGD)

While adolescents suggested clinical treatment of obesity, they also acknowledged that people need to be better educated on obesity and its effects, with this education extending to adolescents’ families and households. It speaks to providing adolescents with skills and opportunity for self-efficacy. The idea to include families may refer to the ability of adolescents to influence their immediate environment, thereby resulting in reduced levels of obesity.

“ … have this mindset of teaching people about obesity and telling them that being obese can cause diseases as I believe that lot of people are very scared of being sick … “(P6_all male FGD) … share the information with our brothers, sisters, and everyone at home (P7_all male FGD)

Rural South African adolescents’ knowledge of obesity is complex, and adolescents recognise multiple factors or pathways as they relate to their general views and causes of obesity. Adolescents view medical treatment to intervene with obesity.

## Discussion

This research has highlighted, firstly, that rural South African adolescents have some knowledge of the health risk that obesity imposes on an individual, recognising that obesity is a result of multiple factors. However, the knowledge does not appear to translate into changes in attitude or behaviour as commonly noted in the literature. Secondly, adolescents in this context do not see themselves as agents of behaviour change or even view themselves as having a direct impact on their behaviour as it relates to obesity, but instead see the solutions to obesity as external by highlighting the use of substances, medications, and institutionalisation as possible ways of dealing with obesity.

Adolescents’ views towards obesity were conflicted with both positive and negative opinions expressed. The positive views expressed correlate with a long-standing perception in SA and other African countries that larger women are considered desirable, as size represents happiness, beauty, and the absence of illness [19,32,33]. A study among 10–19 year-old black adolescent females living in an urban South African setting showed that there is still a desire for a higher BMI [34–36]. However, negative views expressed by adolescents conflicted with these positive views. This has been seen in the previous literature, where some adolescents expressed that thinness is ideal – a belief often found on social media [37]. These competing views have also been found amongst urban adolescents in SA [34]. Amongst the negative views of obesity among adolescents is the belief that obesity is a sign of laziness, found in other studies in varying contexts [38]. These conflicting views may have implications for interventions focused on obesity. The opposing views should be acknowledged and an understanding where they come from understood so that they can be addressed in interventions.

The cultural belief that a bigger body is better preceded the HIV pandemic [32,38–42]. However, the cultural belief was further fuelled by the pandemic and the perception that thinness equated to sickness in the presence of HIV. This shaped the cultural beliefs of South Africans and led to higher body weight being seen as healthy and symbolising happiness [43,44]. Interestingly, in this study, adolescents identified HIV treatment as a possible cause of obesity, whereas historically, HIV/AIDS was described as the ‘wasting’ disease, with weight loss viewed as a marker of disease progression [45]. In recent years, it has been noted that a significant proportion of women initiated in new first line ART regimen do gain weight [44,46–49], not just gaining the weight that they have lost but also gaining additional weight [50]. The findings from this study highlight a clear shift in the perception of HIV and obesity: from HIV as a disease of wasting, to HIV and its treatment, as a disease of obesity. As a result of these transitioning views, shared not only by adolescents, but adults in urban Soweto as well [36], there now may be HIV stigma associated with obese people who may not be HIV positive, the same type of HIV stigma that thin people previously experienced. Our study did not specifically explore how adolescents’ views on the association between HIV and obesity developed. However, the association has shifted cultural understanding of obesity as a possible ‘tell’ for being on HIV treatment. This may have implications for interventions for both HIV and obesity amongst rural South African adolescents.

In combating obesity, adolescents suggested: (i) the closure of local community vendors, (ii) the use of medications or hospitalization of those who are obese and (iii) the use of education for adolescents and their families. Adolescents suggested social and health system interventions and more inclusive education about obesity for their families as part of the treatment. This highlights the importance of the bidirectionality of personal, environmental, and cognitive factors in addressing obesity in this context. While adolescents have the knowledge and recognise, to a degree, various environmental factors that could cause a change in their behaviour, they do not put themselves directly as agents of that change. This suggests that interventions need to address cognitive, personal, and environmental factors to effect change.

Adolescents suggested that community fast-food vendors could be closed to reduce access to unhealthy foods and so address obesity in the community. With the socio-economic transition underway in urban and rural South Africa, there has been an increase of easily accessible informal street vendors selling low-cost fast foods and snacks [32,51], which adolescents frequent with their peers [52]. A study from Cape Town highlighted that local shops lacked healthy food choices such as fruits and vegetables and unhealthy foods were more readily available in many of these local shops. Other studies have recommended that efforts be made to encourage local food shops to stock healthy food items [53]. The closing down of the shops as suggested by adolescents may not be feasible given the economic hardship in these communities; however, it is important to consider and address the foods sold in the shops that are accessible to adolescents.

Adolescents suggested hospitalization or medications as an avenue to address obesity. This indicates a general lack in understanding of community-based approaches to addressing obesity and shifted the responsibility away from the individual. Previous research, including work from SA, suggests that behaviour change approaches, which are considered the cornerstone of health promotion interventions, should be conducted and if those fail, then weight loss medication and/or surgery can be considered [54,55]. Our study did not specifically ask which obese people adolescents considered needing hospitalisation. That may be an important aspect to consider as it may speak to what boundaries adolescents consider as obese, and perhaps these will not be the more biomedical cut-offs that are traditionally used to define overweight and obesity.

Our research suggests that adolescents felt education on obesity is not just important for themselves but for their families as well. This finding highlights the importance of not only intervening at an individual level but also targeting households (environmental factors), leading to buy-in from the family which may enhance the impact of interventions targeted at reducing obesity amongst adolescents. The study findings have implications on the types of interventions, particularly much needed community-based interventions to combat obesity. There are also potential future societal and policy implications as the development of community-based interventions may ultimately have an indirect impact on health care utilisation, health care resources and clinical practice, particularly related to non-communicable diseases consequent on obesity.

In SA, intervention studies in recent years have focused on specific aspects of reducing the burden of obesity in adolescents. ‘HealthKick’, a school-based intervention, comprised diet and physical activity interventions aimed at preventing overweight in children and adolescents and thereby reducing the risk of chronic diseases such as Type 2 diabetes mellitus. The study identified significant improvement in self-efficacy and nutrition knowledge but not in children’s dietary behaviour [56]. Notable challenges included lack of parental involvement, time and available resources, poor physical environment at schools and socio-economic considerations within households. The study concluded that increased parental involvement and greater support from schools were important aspects to consider for future interventions. A study underway in KwaZulu-Natal [57] highlights the importance of not only focusing on adolescents’ attitudes, behaviours and choices, but on how different individual, social and societal aspects may influence adolescents’ decisions. Understanding how adolescent’s behavioural practices are formed, and the process of leading to rejecting or accepting behaviours is likely to be important to developing impactful, contextually relevant interventions aimed at mitigating obesity among adolescents.

Applying SCT to the development of adolescent behaviour change interventions in context of our findings, would suggest: (i) the adolescents need to understand that they are both agents for change and responders to change; (ii) adolescents (individually and with peers) and their families need to be educated separately and together on obesity in order to influence behaviour change in their environment enabling change in social norms; and (iii) adolescents need to be empowered to know that they are in control of their own behaviour and need to be supported through the process of change.

## Strengths and limitations

The study has provided insights into adolescents’ understanding and views on obesity, which is one of the strengths of this study. The study explored these views in a rural context. However, this study may not be generalizable to a broader adolescent population. In our study, while we explored the understanding of obesity amongst rural South African adolescents, we did not ask adolescents directly about the sources of their information. Where adolescents get their information may be valuable in the design and targeting of future interventions. As in all research, there is also the potential for researcher bias, which we attempted to mitigate through iterative development of the interview guides and independent coding of the transcripts. The SCT has only been used in the findings and could be a limitation. A recommendation would be that future studies incorporate the SCT from the beginning, including development of the interview guide.

## Conclusion

Findings from this research provide rural South African adolescents’ perspectives of the causes and consequences of obesity and propose their solutions to reduce obesity in their community. Adolescents appear to understand the causes and health consequences of obesity; however, they do not associate current health behaviours to direct risk of disease later in life. Adolescents have a ‘*medicalised*’ view of treatment for obesity or blame environmental factors, and do not view themselves as agents of their own behaviour change. Interventions aimed at changing adolescents’ views on personal behaviour, promoting healthy lifestyle choices, and mitigating environmental factors so as to allow health choices to be made are needed to mitigate the risk of obesity in rural SA, and formative work, such as this work, is essential to tailor interventions to adolescents’ knowledge and views of obesity.

## Paper context

Obesity is a global pandemic and South Africa is no exception. Limited understanding exists of the views and knowledge of obesity amongst rural South African adolescents. Using the social cognitive theory, this research highlights the conflicting views and multifactorial factors associated with obesity and identifies possible ways of addressing obesity in this context. The study highlights the importance of historic and contextual considerations when developing interventions to mitigate adolescent obesity.
